# Artificial Neural Network Algorithms to Predict Resting Energy Expenditure in Critically Ill Children

**DOI:** 10.3390/nu13113797

**Published:** 2021-10-26

**Authors:** Giulia C. I. Spolidoro, Veronica D’Oria, Valentina De Cosmi, Gregorio Paolo Milani, Alessandra Mazzocchi, Alireza Akhondi-Asl, Nilesh M. Mehta, Carlo Agostoni, Edoardo Calderini, Enzo Grossi

**Affiliations:** 1 Department of Clinical Sciences and Community Health, University of Milan, 20122 Milan, Italy; giulia.spolidoro@unimi.it (G.C.I.S.); valentina.decosmi@unimi.it (V.D.C.); gregorio.milani@unimi.it (G.P.M.); 2 Fondazione IRCCS Ca’ Granda Ospedale Maggiore Policlinico, Anestesia e Terapia Intensiva Donna-Bambino, 20122 Milan, Italy; veronica.doria@policlinico.mi.it (V.D.); edoardo.calderini@policlinico.mi.it (E.C.); 3 Pediatric Intermediate Care Unit, Fondazione IRCCS Ca’ Granda Ospedale Maggiore Policlinico, 20122 Milan, Italy; alessandra.mazzocchi1@gmail.com; 4 Pediatric Unit, Fondazione IRCCS Ca’ Granda Ospedale Maggiore Policlinico, 20122 Milan, Italy; 5 Department of Anesthesiology, Critical Care and Pain Medicine, Boston Children’s Hospital, Boston, MA 02115, USA; Alireza.Akhondi-Asl@childrens.harvard.edu (A.A.-A.); Nilesh.Mehta@childrens.harvard.edu (N.M.M.); 6 Center for Nutrition, Boston Children’s Hospital, Boston, MA 02115, USA; 7 Department of Anaesthesia, Harvard Medical School, Boston, MA 02115, USA; 8 Villa Santa Maria Foundation, Neuropsychiatric Rehabilitation Center, Autism Unit, 22038 Tavernerio, Italy; enzo.grossi@bracco.com

**Keywords:** energy expenditure, metabolism, nutrition, children, pediatrics, critical care, pediatric intensive care, neural networks

## Abstract

Introduction: Accurate assessment of resting energy expenditure (REE) can guide optimal nutritional prescription in critically ill children. Indirect calorimetry (IC) is the gold standard for REE measurement, but its use is limited. Alternatively, REE estimates by predictive equations/formulae are often inaccurate. Recently, predicting REE with artificial neural networks (ANN) was found to be accurate in healthy children. We aimed to investigate the role of ANN in predicting REE in critically ill children and to compare the accuracy with common equations/formulae. Study methods: We enrolled 257 critically ill children. Nutritional status/vital signs/biochemical values were recorded. We used IC to measure REE. Commonly employed equations/formulae and the VCO_2_-based Mehta equation were estimated. ANN analysis to predict REE was conducted, employing the TWIST system. Results: ANN considered demographic/anthropometric data to model REE. The predictive model was good (accuracy 75.6%; R^2^ = 0.71) but not better than Talbot tables for weight. After adding vital signs/biochemical values, the model became superior to all equations/formulae (accuracy 82.3%, R^2^ = 0.80) and comparable to the Mehta equation. Including IC-measured VCO_2_ increased the accuracy to 89.6%, superior to the Mehta equation. Conclusions: We described the accuracy of REE prediction using models that include demographic/anthropometric/clinical/metabolic variables. ANN may represent a reliable option for REE estimation, overcoming the inaccuracies of traditional predictive equations/formulae.

## 1. Introduction

A high metabolic variability may impact nutrition requirements for critically ill patients, particularly children. Accordingly, energy requirements are not stable throughout the course of hospitalization, as they may depend on the medical and pharmacologic interventions (exogenous variables) on the one hand, and the individual metabolic response to inflammation (endogenous variables) and physiologic variables on the other [1]. Accurate estimation of energy requirements is the starting point to define patients’ nutritional needs and it is based on the assessment of energy expenditure. There is universal agreement that the calculation of energy expenditure starts from the assessment of resting energy expenditure (REE), adjusted (in non-critical conditions) for physical activity levels [2]. Indirect calorimetry (IC) provides an accurate measurement of REE by assessing patients’ respiratory gas exchange and converting oxygen consumption (VO_2_) and carbon dioxide production (VCO_2_) into a caloric equivalent with the modified Weir equation [3]. Although IC is the gold standard for REE measurement, it is often unavailable in most pediatric ICUs (PICUs). Within a recent survey, only 14% of PICUs have resources to use IC and, accordingly, nutritional targets for macronutrients, corrected for age/weight, may widely vary too [4].

In the absence of IC, most dietitians use the REE predictive equations to define energy needs and dietary prescriptions, which may often under- or overestimate energy needs, respectively, in critically ill children [5]. The associated energy imbalance may accumulate over time, with deterioration of nutritional status and negative impacts on patients’ outcomes, carrying a higher risk of nosocomial infections along with longer mechanical ventilation and a longer LOS, as well as lower survival rates [4]. Resulting metabolic unbalances in ICU patients, such as blood glucose instability and related consequences, are well recognized [6]. Artificial neural networks (ANN) might represent a more precise and accurate method to estimate REE [7]. ANN are computerized algorithms resembling interactive processes of the human brain allowing for the definition of very complex non-linear phenomena, such as biological systems [8]. The aim of this study was to describe the accuracy of ANN algorithms (ANNs) for the estimation of REE compared to measured REE by IC in critically ill pediatric patients. We also aimed to compare the accuracy of the ANN-derived REE with REE estimated from the most commonly employed estimation formulae.

## 2. Methods

### 2.1. Study Design and Study Population

In this single-center study, all data were consecutively collected in the context of a cross-sectional prospective study [5,9]. For ANN analysis, data were evaluated retrospectively (post-hoc analysis). We enrolled patients consecutively admitted to a 6-bed PICU of a tertiary children’s hospital (Fondazione IRCCS Ca’ Granda, Ospedale Maggiore Policlinico, Milan, Italy) from May 2013 to December 2019. The study was approved by the Ethical Committee of the Policlinico of Milan Hospital (Project identification code 135/2013) and informed consent was obtained.

### 2.2. Nutritional Status and Clinical Characteristics

A multidisciplinary team completed the nutritional assessment and the anthropometric measurements during the hospital stay. Weight (using a gram scale, accurate to 0.1 kg) and length with a 417 SECA stadiometer (^®^ SECA Medical Measuring Systems and scales, Birmingham UK) or a flexible but non-stretchable tape measure were recorded. Body mass index (BMI) was derived (kg/m^2^). Z-scores for weight for age (WFA), BMI for age, weight for length/height (WFL or WFH), and length/height for age (LFA or HFA) were calculated using the WHO Anthro and Anthro Plus ^®^ software, and the WHO reference charts [10]. Stunting (i.e., chronic undernutrition) was diagnosed according to the WHO criteria as LFA (or HFA) z-score < −2. Wasting (i.e., acute undernutrition) was diagnosed according to WHO criteria as WFL (or WFH) z-score < −1 (mild), < −2 (moderate), or < −3 (severe) for children younger than 5 years and as BMI z score < −1 (mild), < −2 (moderate), or < −3 (severe) for children older than or equal to 5 years old. Overweight was defined as WFL (or WFH) z-score > 2 (for children <5 years), and as BMI z score > 1 (for children ≥ 5 years). Obesity was defined as WFL z score > 3 (for children <5 years), and as BMI z score > 2 (for children ≥ 5 years). The REE was measured in thermoneutral conditions using an open-circuit IC (Vmax 29^®^, Sensor Medics, Yorba Linda, CA, USA). VO_2_ and VCO_2_ were measured in spontaneously breathing (canopy mode) and mechanically ventilated (ventilation mode) children for a period of 30 min. Respiratory quotient (RQ) was calculated as VCO_2_/VO_2_ and REE using the modified Weir formula, not accounting for urinary nitrogen excretion [11]. Steady state conditions were defined as at least 5 min with less than 5% variation in RQ, less than 10% variation in VO_2_ and in VCO_2_, and less than 10% variation in minute ventilation. Data from patients who did not meet steady state or had an RQ < 0.67 or > 1.3 were excluded. Energy expenditure was estimated using the following predictive equations/formulae: Harris–Benedict, Harris–Benedict for infants, Schofield for weight, Schofield for weight and height, Oxford for weight, Oxford for weight and height, WHO/FAO/UNU, Talbot tables for weight, Talbot tables for height, and the Mehta equation [12,13,14,15,16,17].

The Mehta equation was calculated only in mechanically ventilated children as it has been validated in this population [17,18]. Clinical characteristics, vital signs (heart rate, blood pressure systolic and diastolic, oxygen saturation—SatO_2_%, respiratory rate, and body temperature °C) and blood values, such as hemoglobin (Hb, g/dL), C-reactive protein (CRP, mg/dL), albumin (g/dL), and blood glucose (mg/dL), were included in the database. Blood concentrations were measured directly after blood sampling, with methods standardized in the central laboratory of the hospital, when the patient entered the study. Clinical characteristics and anthropometric measures were recorded upon admission. Blood tests were performed on the day of the exam. Vital signs were recorded during the IC exam.

### 2.3. Modelling of REE with Artificial Neural Networks

#### 2.3.1. Data Pre-Processing

Our database included 49 variables, among all basic demographic and anthropometric characteristics (gender, male/female; ethnic origin, Caucasian/Asian/South American/African; age; weight; z-score WFA; height; z-score HFA; z-score WFH; BMI, z-score BMI), nutritional status (normal weight, overweight, obesity, stunting, wasting—no, mild, moderate, severe), outcome variables (diagnosis, comorbidities, presence of mechanical ventilation, length of stay, gestational age, weight at birth, current therapy, current nutrition), vital signs (body temperature; heart rate; blood pressure, systolic and diastolic; respiratory rate; oxygen saturation), and some blood values (albumin, hemoglobin, blood glucose, C-reactive protein, aspartate aminotransferase, alanine aminotransferase, blood creatinine, blood calcium, blood phosphate, alkaline phosphatase, serum iron, ferritin, transferrin). Variables presenting at least one missing data were excluded from the ANN analysis. For basic demographic and anthropometric data, we only missed data for z-score WFH and z-score WFA as the World Health Organization provides z-score charts only up to 5 years of age for z-score WFH and up to 10 years of age for z-score WFA (84 and 42 “missing” data, respectively). Regarding outcome variables, we missed complete data for the two variables weight at birth and gestational age (missing data, 79 and 54, respectively). Moreover, we excluded length of stay (LOS) from the ANN analysis as we wanted to provide a predictive algorithm that could be applied by clinicians during PICU hospitalization. Therefore, the inclusion of LOS among the variables would not have provided useful information for a timely REE prediction. For practical reasons, we decided to exclude the variables describing the diagnosis and comorbidities of the patients as this would have required careful categorization of patients’ diagnosis and comorbidities in different disease-based clusters. Since our goal was to provide an algorithm that could be used by clinicians and given that different clinicians may categorize diseases and comorbidities with some slight but relevant differences, we decided to exclude these variables from further ANN analysis to avoid difficulties associated with categorization replicability. For the same reason, we excluded “current therapy” and “current nutrition” as it would have been difficult to categorize drugs and feeding clinical approach for ANN analysis. Finally, our original database presented missing data among all the blood values and vital sign variables. The reason for the unavailability of these data is that, depending on the subject’s condition, clinicians may decide to request different blood exams. Moreover, vital signs may not have always been recorded in the patient’s diary for different reasons (e.g., the sensor was teared away by the patient, vital sign was assessed by the clinician but not recorded in the patient’s diary).

#### 2.3.2. Data Set Analysis

A physician expert in ANN analysis conducted the modelling and the data set analysis. The original data set (data set 1) included the 24 variables for which we had no missing data: mechanically ventilated, gender (male/female) ethnic origin (Caucasian, Asian, South American, African), age, weight, height, BMI, BMI z-score, height for age z-score, normal weight, overweight, obese, wasting (absent, mild, moderate, severe), stunting, VO_2_, VCO_2_, and RQ. Multivariate analysis was carried out with supervised ANN, according to the method previously described [19]. Five different approaches were used. First, the analysis was applied to all 24 variables, including the gas values VO_2_, VCO_2_, and RQ, which were obtained with IC. The reason for developing a model with all variables, gas values included, was purely technical, with the aim of obtaining a “baseline” predictive model developed under the best possible conditions, i.e., with the inclusion of gas exchange monitoring. The system was then tested on four different variants of the data set, the first using a data set with 21 variables, avoiding all gas values (VO_2_, VO_2_, RQ), the second including the 21 variables and VO_2_, the third including the 21 variables and VCO_2_, and the fourth including the 21 variables and RQ. This was done with the purpose of better defining the contribution of each gas value to the accuracy of prediction.

In a subgroup of children, it was possible to extend the analysis to include some additional endogenous and physiological variables. This extended data set (data set 2) included less subjects but more variables (32 variables) with complete data for each subject: the 24 variables mentioned above and 8 “functional” inputs hereby listed, that is, heart rate, blood pressure (systolic and diastolic), SatO_2_, and body temperature, as well as CRP, Hb, and blood glucose. We chose to include all vital signs except for respiratory rate and only CRP, Hb, and blood glucose, among the blood values, because this combination allowed us to both add more information on patients’ clinical/functional status while keeping an acceptable number of subjects to perform ANN analysis. The inclusion of functional inputs was aimed at testing the hypothesis of a more accurate estimation of REE during the critical state beyond basic demographic and anthropometric data. Accordingly, the inclusion of variables capable of describing modification of the functional status might help to improve ANN model prediction. As for the original data set, the model was first developed considering all the variables, with the scope of obtaining a baseline model. The modelling was then tested on a 29-variable data set, without gas values, and then on a 30-variable data set, also including VCO_2_, which can be measured by new-generation ventilators or by capnography with meaningful clinical relevance.

#### 2.3.3. TWIST (Training with Input Selection and Testing) System

In order to cut down non-relevant variables in the database (i.e., the variables not carrying meaningful additive information for the prediction task), which cause a loss in the power of our inferences, we employed a special ‘artificial organism’ called TWIST, suitably designed for sorting out the most relevant variables for the sake of prediction/classification [20]. The TWIST system consists of a combination of two systems, training/testing (T&T) and input selection (IS), respectively. The T&T system is a robust data re-sampling technique that is able to arrange the source sample into sub-samples, all of which possess a similar probability density function. In this way, the database was split into two or more sub-samples in order to train, test, and validate the ANN models as effectively as possible on the basis of the available data. The IS system is an evolutionary ‘wrapper’ system that selects variables in order to minimize their number while preserving the actual amount of task-relevant information contained in the data set. The combined action of these two systems allowed us to substantially increase the inferential power of our ANN system, while circumventing, at the same time, a few major technical issues. Both systems are based on a genetic algorithm, the Genetic Doping Algorithm (GenD) developed at Semeion Research Centre (Rome, Italy) [21].

The TWIST pre-processing singles out the variables that prove to be most significant for the prediction/classification task, while producing, at the same time, the training set and the testing set, which are extracted from a probability distribution very close to the one that provided the best performance in the task. On the variables selected by the TWIST system, the functional approximation/prediction task is carried out by means of a supervised multi-layer perceptron, with four hidden units. The study sample was randomly divided into two main sub-samples: the training set sub-sample and the testing sub-sample. Training and testing sets were then reversed, and consequently, for each record of the data set, a blind prediction was carried out. The accuracy results were expressed as the average of the results obtained in the two independent testing sets.

### 2.4. Statistical Analysis

The REE value predicted by ANN was compared with the REE measured with IC by univariate linear regression. The mean absolute error (MAE), i.e., the mean of the absolute difference between the predicted and actual value, and the mean relative error, i.e., the ratio of the MAE of the measurement to the actual measurement, the Pearson coefficient of determination (R^2^), and the F-test for two sample analyses of variance were used to measure the predictive accuracy of ANN, when appropriate. Data are given as mean and standard deviation, absolute, or percentile values. Significance was assumed when *p* < 0.001 taking into account the existence of multiple tests. Analyses were performed using SPSS 20.0 (Statistical Package for Social Science. Inc., Armonk, NY, USA). The same fitting was carried out with all the equations/formulae on study.

## 3. Results

### 3.1. Data Set 1

#### 3.1.1. Population Characteristics

The whole population of the original data set (data set 1) consisted of 257 pediatric patients (145 males, 56.4%) of whom, 102 (39.5%) were mechanically ventilated. Their characteristics are shown in Table 1.

#### 3.1.2. Linear Correlations

**Figure 1:**
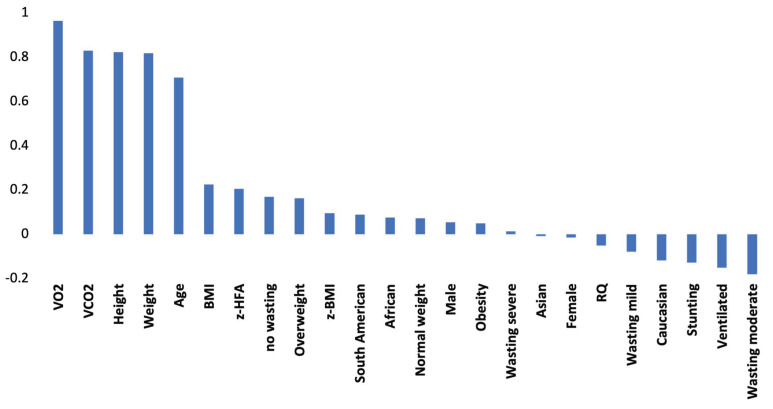
Correlations between the study variables and the REE value. Abbreviations: VO_2_ = Oxygen Consumption; VCO_2_ = Carbone Dioxide Production; RQ = Respiratory Quotient; BMI = Body Mass Index; z-BMI = z-score BMI; z-HFA = z-score Height for Age.

Figure 1 shows the linear correlation values between the study variables and the REE value. As expected, VO_2_, VCO_2_, height, weight, and age were highly correlated with REE. In any case, the absolute value of Pearson R of the other variables was rather low, and this offers a further rationale for the application of ANN, especially when avoiding gas values.

#### 3.1.3. Fitting of REE with the Equations

**Figure 2:**
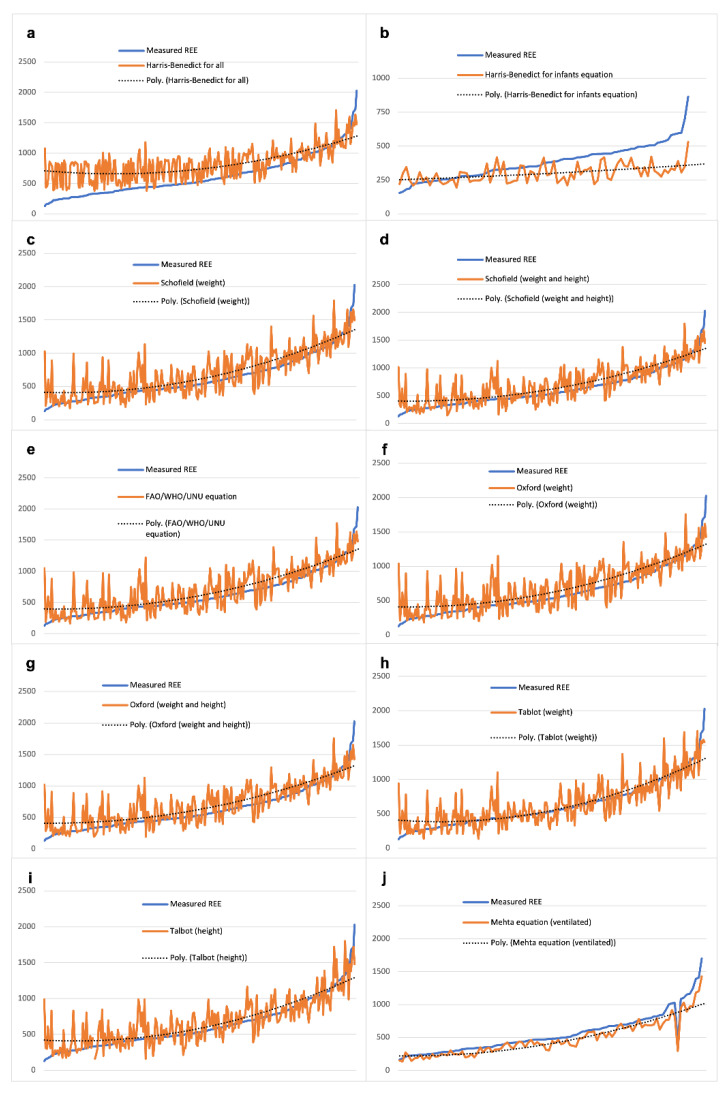
Real REE approximation with predictive equations. Legend Harris–Benedict for all (**a**), Harris–Benedict for infants (**b**), Schofield (weight) (**c**), Schofield (weight and height) (**d**), WHO/FAO/UNU equation (**e**), Oxford (weight) (**f**), Oxford (weight and height) (**g**), Talbot (weight) (**h**), Talbot (height) (**i**), Mehta equation in ventilated children (**j**).

Figure 2 show the real REE approximation obtained with all the equation/formulae considered in the study. The blue line expresses the true REE values, the orange line is the corresponding fitting of the method under evaluation, and the dotted line is the tendency line of the method described by polynomial equations.

All the equations, with the exception of Mehta’s, appear to systematically overestimate the true REE value and mostly in the left side where true REE reaches the lowest values. The contrary is observed at the extreme right of the graphic, where true REE evaluations skip over the estimated values (that is, this quite restricted cue may be under-estimated). The most meaningful differences are displayed by the Harris–Benedict equations, even more after adjusting for age <12 months.

#### 3.1.4. Fitting of REE with Artificial Neural Networks: Baseline Analysis (24 variables)

**Figure 3:**
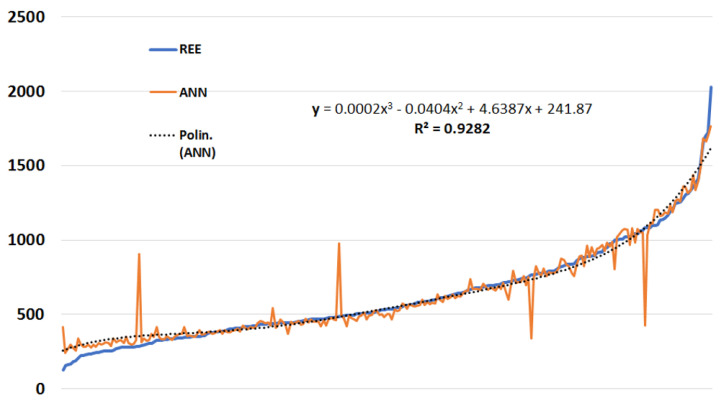
Real REE approximation with neural networks. Legend. Blue line expresses the true REE values; orange line represents the corresponding fitting of the method under evaluation, and the dotted line is the tendency line described by a five-degree polynomial equation. REE = Resting Energy Expenditure; ANN = Artificial Neural Networks.

The TWIST^®^ system selected the following seven variables carrying the maximal amount of information to build up a predictive model: gender (female, male), weight, BMI, VO_2_, VCO_2_, and RQ. The final model, based on these seven variables, expressed a functional approximation of the actual REE value within a protocol based on a bipartite division of the data set between a training set sub-sample (*n* = 125) and a testing sub-sample (*n* = 132). Training and testing sets were then reversed, and consequently, for each record of the data set, a blind prediction was carried out. Within this approach, the neural network tendency line appears to be almost superimposed on the true REE values curve (Figure 3).

#### 3.1.5. Comparative Statistics between Tests on Study

The modelling obtained by the ANN reached an average absolute error of 38.1 calories (93.9% accuracy) with an R^2^ = 0.928. The comparative values obtained with the other equations were less precise. The lowest absolute error resulted from the Mehta equation (which also requires VCO_2_), that is, 89.7 calories (84.0% accuracy), followed by the Talbot table for weight with an average absolute error of 142.3 calories (77.2% accuracy). The Harris–Benedict equation showed, on the other hand, an average absolute error of 244.2 calories (60.8% accuracy) (Table 2).

#### 3.1.6. ANN Analysis to Evaluate the Contribution Given by Gas Values to REE Fitting

We hereby describe the application of the TWIST system to five differential analyses, the first including all gas values VO_2_, VCO_2_, and RQ (24 variables); the second avoiding all gas values (21 variables); the third avoiding VCO_2_ and RQ and maintaining VO_2_; the fourth avoiding VO_2_ and RQ while maintaining VCO_2_; and the fifth avoiding VO_2_ and VCO_2_ while maintaining RQ (22 variables each).

Table 3 shows the variables selected for the modelling, the predictive results obtained by ANN, and the comparison with the results obtained in the original (baseline) data set.

### 3.2. Data Set 2

#### 3.2.1. Population Characteristics

The purpose of data set 2 was to provide more functional inputs to the ANN predictive model. Functional parameters were included in the original data set 1 but not for all subjects. Therefore, the population for data set 2 was reduced to 199 pediatric patients (112 males, 56.3%), of whom 93 (46.7%) were mechanically ventilated. Patients’ characteristics, including vital signs and blood values, are presented in Table 4.

#### 3.2.2. Linear Correlations

**Figure 4:**
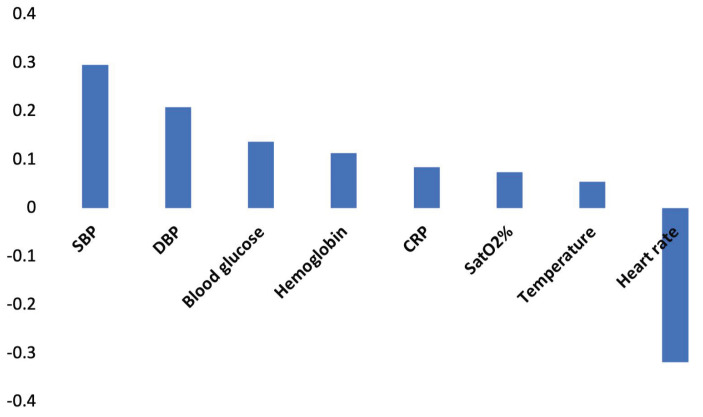
Correlations between the study variables added in Data set 2 and the REE value. Abbreviations: SBP = Systolic Blood Pressure; DBP = Diastolic Blood Pressure; CRP = C-reactive protein; SatO_2_% = Oxygen Saturation (%).

The linear correlations between the study variables and the REE values were very similar to data set 1 (Supplementary Materials, Additional File S1). The gas values (VO_2_, VCO_2_), height, weight, and age were highly correlated with REE. In all other cases, the Pearson R value of the other variables was low. Figure 4 shows the correlations between the functional values added in data set 2 and REE.

#### 3.2.3. Real REE Approximation with Artificial Neural Networks

**Figure 5:**
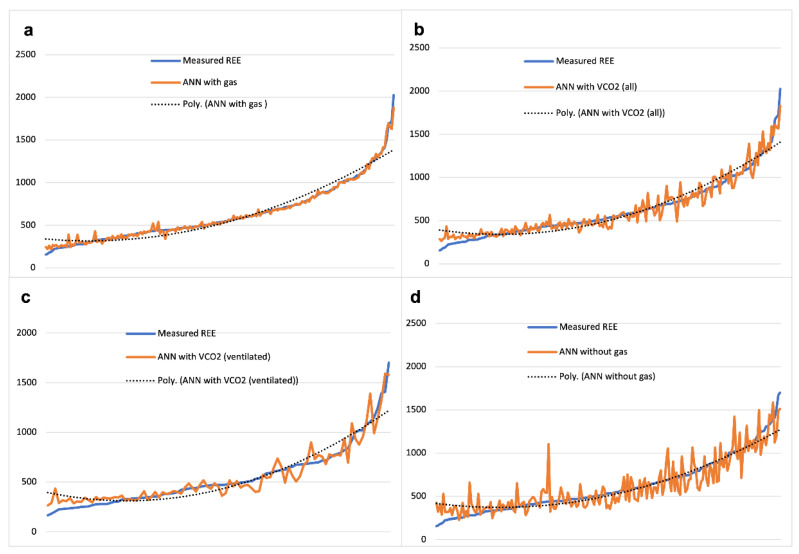
Data set 2 real REE approximation with ANN. Legend. ANN with gas (**a**), ANN with VCO_2_ (all subjects) (**b**), ANN with VCO_2_ (ventilated subjects) (**c**), ANN without gas (**d**). Abbreviations: REE = Resting Energy Expenditure; ANN = Artificial Neural Networks; VCO_2_ = Carbone Dioxide Production.

The inclusion of functional variables in data set 2 improved the prediction values of REE by ANN. Figure 5 shows the real REE approximation with ANN best to worse, respectively. The neural network trend of the baseline model developed with the addition of the gas values appears to be almost superimposed on the true REE values curve. The model developed with no gas values fits less, while the VCO_2_ models stand somewhere in between the two. Real REE approximation by the predictive equations/formulae is not visually represented for data set 2, but it was comparatively worse than ANN modelling and similar to the findings in Figure 2 (Supplementary Materials, Additional File S2).

#### 3.2.4. Comparative Statistics between All Methods on Study

All the methods explored for the prediction of REE are displayed in Table 5. As anticipated, the inclusion of functional inputs in data set 2 provided an advantage in terms of the performance of the ANN models. The best prediction of REE was obtained with the ANN baseline model (with gas), reaching an average absolute error of 23.3 calories (96.3% accuracy) with an R^2^ = 0.968. The comparative values obtained with the other fitting methods were less precise. The ANN model with VCO_2_ and the Mehta equation (which also requires VCO_2_) followed as the second-best method for REE estimation in terms of absolute error. The ANN model developed without gas values fitted less but was still better than the remaining equations/formulae in the study. The inclusion of functional parameters among the inputs indeed improved the model. The Talbot table for weight without gases ranked as the second predictor, with an average absolute error of 132.7 calories (79.0% accuracy). The Harris–Benedict equation was seemingly the worse option, with an average absolute error of 245.4 calories (61.2% accuracy).

#### 3.2.5. ANN Analysis to Evaluate the Contribution Given by Gas Values to REE Fitting

We hereby describe the application of the TWIST system to three differential analyses, the first including all gas values VO_2_, VCO_2_, and RQ (32 variables); the second avoiding all gas values (29 variables); and the third avoiding VO_2_ and RQ while maintaining only VCO_2_ (30 variables).

Table 6 shows the variables selected for the modelling, the predictive results obtained by ANN, and the comparison with the results obtained in the original (baseline) data set.

## 4. Discussion

We have described for the first time an ANN-based REE predictive model to offer insight into the potential of machine learning to provide a valid and accurate REE prediction in critically ill children. Machine learning may offer a new opportunity to explore the complexity of metabolic changes in different physiologic and pathologic conditions [22]. The use of ANN models to predict REE has been found to be reliable in healthy children and adults, including obese patients [7,23]. In our current study, we further described the feasibility of employing ANN models to predict REE in critically ill children.

Our results highlight variables that are relevant to REE prediction. The TWIST system selected VO_2_ and VCO_2_, RQ, weight, BMI, and gender (female, male) as meaningful variables. The result is consistent with the fact that VO_2_ and VCO_2_ are used to compute the modified Weir equation to obtain REE with IC, hence providing high concordance due to mathematical coupling. Moreover, height, weight, and gender are among the variables taken into account to estimate REE by the most commonly employed predictive equations/formulae, including the Schofield equation, Harris–Benedict equation, Talbot tables, and many others. The model developed by considering all the gas values (VO_2_, VCO_2_, RQ) to predict REE was therefore the most accurate and was used as a baseline to understand how accurate ANN can be compared to the modified Weir equation.

To better appreciate the contribution given by each gas value to the REE fitting model, we further applied the TWIST system to four additional variants of the same data set, the first avoiding all gas values VCO_2_, VO_2_, and RQ (21 variables); the second including only VO_2_; the third including only VCO_2_; and the fourth maintaining only RQ (22 variables each). When removing VO_2_, VCO_2_, and RQ altogether, the accuracy of the REE predictive model was similar to most predictive equations/formulae. The Talbot table for weight prediction correlated better and was slightly more accurate than the model. The Harris–Benedict equation was the least accurate of all. The accuracy of the model did not considerably improve when RQ was included in the data set analysis. Instead, the inclusion of either VO_2_ or VCO_2_ in the data set sensibly improved the accuracy of the REE predictive model. In both cases, the accuracy was superior to all the predictive equations/formulae considered for the study. Furthermore, the model developed including VO_2_ was almost as good as the one developed considering all gas exchange variables (baseline). This finding may be relevant from a more theoretical/physiological perspective, as it may indicate that oxygen consumption is more relevant than carbon dioxide production in defining REE. However, a VO_2_-based predictive model would not be useful in clinical practice, since VO_2_ is not commonly measured in the hospital setting, unless IC is performed. On the other hand, a VCO_2_-based predictive model would be even more valuable in the critical care setting, as VCO_2_ may be monitored in ventilated patients using capnography or even better, using new-generation ventilators, which includes VCO_2_ monitoring among their functions. The opportunity to use VCO_2_ in a predictive algorithm has already been explored by Mehta et al. and Kerklaan et al., who developed and validated a VCO_2_-based predictive equation (the Mehta equation) [17,18]. In the present analysis, the accuracy of the VCO_2_ predictive model was equal to the accuracy of the Mehta equation.

Our results should not be considered as supportive of replacing IC, which remains the true gold standard for assessing REE in PICU. Evidently, basic demographic and anthropometric parameters alone do not provide sufficient information to allow an accurate prediction of REE with machine learning. Compared to healthy and obese children, a more complex and comprehensive metabolic monitoring is needed during acute illness to accurately assess REE [7]. Our results do suggest that predicting REE with ANN models may represent a better alternative to the common REE estimations when IC is not available in the PICU setting.

Models developed by ANN would be highly improved with the inclusion of variables possibly “marking” the changes from physiology to acute illness. For this reason, in a smaller subset of patients, we were able to include data regarding vital signs and a few blood values in the analysis (physiological and endogenous variables or “functional” inputs). As expected, the inclusion of heart rate, blood pressure (systolic and diastolic), SatO_2_, and body temperature, as well as CRP, Hb, and blood glucose, improved the accuracy of the prediction. The variables selected by TWIST for ANN-based REE prediction were slightly different in the data set 2 analysis compared to data set 1. The explanation could be dual: the number of subjects included was different between the two data sets; moreover, in data set 2, more variables were included. Interestingly, in the model considering all gas values among the functional variables, only oxygen saturation (SatO_2_%) was selected by the TWIST system. The explanation for this could be that VO_2_, VCO_2_, and RQ already fully describe the metabolic state of hospitalized children, without the need for additional endogenous or physiological information. Since SatO_2_ could indicate an imbalance between gas exchanges, selecting this variable could help the system modulate the prediction. For the model not considering gas values and for the model considering only VCO_2_ among the gas values, body temperature, SatO_2_, CRP, and blood glucose, CRP was selected. The fact that in both models, CRP was selected as a meaningful variable is consistent with the notion that CRP is an indicator of illness severity and acute metabolic response and inflammation. The selection of body temperature among the variables for the model with no gas values is an important notion as body temperature importantly influences REE, with studies showing a positive correlation with factors per degree Celsius ranging from 6% to 8% [24]. Finally, the inclusion of blood glucose among the variables selected by TWIST for the VCO_2_ model could be relevant, given that glucose abnormalities (especially hyperglycemia) are common adverse events in the critical care setting, which can result from the endogenous response to acute illness, regulated by different hormones (e.g., glucagon, cortisol, growth hormone, catecholamine, insulin), but could also be influenced by many medications [25].

Our results suggest that machine learning may overcome the classic three of four features of linear combination predictive models on which REE predictive equation/formulae are based, and obtain a more accurate estimation of REE, by improving the number of inputs considered in the predictive model. By applying the TWIST system to different combinations of the same data set, all the models developed were superior to the predictive equations/formulae considered in the study. As expected, the model with all gas values (baseline model) was the most accurate. The model developed without gas values was less accurate but still showed good accuracy for clinical practice. The VCO_2_ model reached a very high degree of accuracy (close to 90%). The model was even more accurate than the Mehta equation, possibly suggesting a refinement of REE prediction based on VCO_2_. In any case, these findings need to be confirmed in clinical practice by testing the model on VCO_2_ values actually measured with capnography and/or by ventilators.

The current study has some limitations. Since these data were analyzed as part of a post-hoc analysis, we were unable to include some variables that could have added useful information to our model. For instance, we did not have a recorded severity of illness score (e.g., Pediatric Risk of mortality Index II, PIM2). Moreover, we had insufficient data to assess the effects of sedation, analgesia, vasoactive drugs, or other pharmacological therapies on patients. Finally, even though blood values and vital signs were collected in the database, many data were missing. Therefore, we chose to include all vital signs except for respiratory rate and only CRP, Hb, and blood glucose, among the blood values, because this combination allowed us to include more functional inputs, while keeping a sufficient number of subjects for the scope of the study.

## 5. Conclusions

The delivery of optimal nutrition to critically ill children relies on accurate assessment of energy needs. Indirect calorimetry, the gold standard for measurement of REE, is not available in most centers. In the absence of IC, machine learning may represent a feasible cost-effective solution to predict REE with good accuracy and therefore a better alternative to the common REE estimations in the PICU setting. We described demographic, anthropometric, clinical, and metabolic variables that are suitable for inclusion in ANN models to estimate REE. The addition of VCO_2_ measurements from routinely available devices to these variables may provide an accurate assessment of REE using machine learning. Further refinement of models using other variables must be tested in larger populations to determine the true role of machine learning in precise individual REE prediction, particularly in critically ill children.

## Figures and Tables

**Table 1:** Anthropometric and metabolic measurements of the study population.

	*N* = 257		
*Demographic*		*Metabolic (Indirect calorimetry)*	
Age, years	2.4 (6.0) ^#^	VO_2_, L/min	0.09 (0.05)
Male	145 (56.4)	VCO_2_, L/min	0.07 (0.04)
*Anthropometric*		RQ	0.77 (0.12)
Weight, kg	15.6 (12.2)	Resting Energy Expenditure, kcal/die	623.3 (325.7)
Height, cm	93.4 (30.5)	*Metabolic (equations/formulae)*	
BMI, kg/m^2^	15.9 (3.2)	REE Harris–Benedict equation	824.3 (260.2)
z-score BMI	−0.7 (2.0)	REE Harris–Benedict equation for infants	964.3 (134.6)
z-score weight for age	−0.9 (1.7)	Schofield (weight) equation	700.9 (347.6)
z-score height for age	−1.2 (1.9)	Schofield (weight and height) equation	703.0 (344.3)
z-score weight for height	−0.6 (2.0)	FAO/WHO/UNU equation	701.4 (353.1)
*Outcomes*		Oxford (weight) equation	703.1 (335.9)
Mechanically ventilated	102 (39.5)	Oxford (weight and height) equation	705.0 (332.8)
Length of PICU stay, days	6.0 (12.0) ^#^	Talbot (weight) equation	650.1 (332.4)
		Talbot (height) equation	675.6 (325.5)
		Mehta equation *	475.6 (257.0)

Abbreviations: BMI = Body Mass Index; PICU = Pediatric Intensive Care Unit; VO_2_ = Oxygen Consumption; VCO_2_ = Carbone Dioxide Production; RQ = Respiratory Quotient; REE = Resting Energy Expenditure; FAO = Food and Agriculture Organization; WHO = World Health Organization; UNU = United Nation University. Data are presented as mean and standard deviation or frequency and percentage. * mechanically ventilated children. ^#^ data are expressed as median and interquartile range (IQR) according to their distribution.

**Table 2:** Fitting performances of true REE by the tests under study.

Overall Group (*N* = 257), Measured REE = 623.3 (325.7)									
FITTING METHOD	Predicted REE		Absolute Error	Accuracy	Relative Error	Accuracy	F-Test Two-Sample		
	Mean	SD	Mean	%	Mean	%	F-Statistic	*p*-Value (Two Tails)	Pearson (R^2^)
ANN with gas (baseline)	651.4	329.0	38.1	93.9	0.058	94.2	0.982	0.881	0.928
Harris–Benedict	824.3	260.2	244.2	60.8	0.610	38.9	1.567	<0.001	0.497
Harris–Benedict for infants	299.5	64.5	103.3	72.8	0.254	74.6	3.739	<0.0001	0.288
Schofield (weight)	700.9	347.6	164.7	73.6	0.351	64.9	0.878	0.298	0.664
Schofield (weight and height)	703.0	344.3	160.8	74.2	0.348	65.2	0.895	0.374	0.671
FAO/WHO/ UNU	701.4	353.1	168.7	72.9	0.358	64.2	0.851	0.196	0.653
Oxford (weight)	703.1	335.9	163.7	73.7	0.352	64.8	0.941	0.624	0.655
Oxford (weight and height)	705.0	332.8	158.7	74.5	0.344	65.6	0.958	0.733	0.671
Talbot (weight)	650.1	332.4	142.3	77.2	0.300	70.0	0.960	0.746	0.691
Talbot (height)	675.6	325.5	147.6	76.3	0.320	68.0	1.002	0.989	0.684
Mehta *	475.6	257.0	89.7	84.0	0.160	84.0	1.380	0.107	0.906

Abbreviations: REE = Resting Energy Expenditure; ANN = Artificial Neural Networks; FAO = Food and Agriculture Organization; WHO = World Health Organization; UNU = United Nation University; SD = standard deviation. * mechanically ventilated children. Measured REE in mechanically ventilated children was 562.3 (301.9).

**Table 3:** Results obtained in the five differential analyses relevant to the role of gas values.

Data Set 1	Baseline	1	2	3	4
Variables number of the data set	24	21	22	22	22
Gas variables	VO_2_; VCO_2_; RQ	none	VO_2_	VCO_2_	RQ
Variables selected by TWIST system	male	female	Weight	African	mechanically ventilated
	female	age	BMI	weight	male
	weight	weight	Obese	height	female
	BMI	height	VO_2_	z-HFA	Asiatic
	VO_2_	z-BMI		overweight	weight
	VCO_2_	z-HFA		Wasting (severe)	height
	RQ	No wasting		VCO_2_	BMI
		wasting (mild)			z-HFA
		wasting (moderate)			normal weight
		wasting (severe)			Wasting (severe)
		stunting			RQ
predictive accuracy	93.9%	75.6%	92.9%	84.4%	78.0%
mean absolute error	38.1	149.1	44.0	96.9	136.8
Person R^2^	0.928	0.713	0.914	0.829	0.701

Abbreviations: TWIST = Training with Input Selection and Testing; VO_2_ = Oxygen Consumption; VCO_2_ = Carbone Dioxide Production; RQ = Respiratory Quotient; Z-BMI = z score for Body Mass Index; z-HFA = z-score Height for Age.

**Table 4:** Anthropometric, functional, and metabolic measurements of the study population.

			*N* = 199
*Demographic*		*Metabolic (Indirect calorimetry)*	
Age, years	2.3 (6.4) ^#^	VO_2_, L/min	0.09 (0.05)
Male	112 (56.3)	VCO_2_, L/min	0.07 (0.04)
*Anthropometric*		RQ	0.75 (0.11)
Weight, kg	16.1 (12.7)	Resting Energy Expenditure, kcal/die	632.3 (339.9)
Height, cm	94.0 (31.1)	*Metabolic (equations/formulae)*	
BMI, kg/m^2^	16.1 (3.4)	REE Harris–Benedict equation	833.5 (262.3)
z-score BMI	−0.6 (2.1)	REE Harris–Benedict equation for infants	718.9 (357.7)
z-score weight for age	−0.9 (1.7)	Schofield (weight) equation	711.5 (353.4)
z-score height for age	−1.1 (1.9)	Schofield (weight and height) equation	712.6 (351.0)
z-score weight for height	−0.6 (2.0)	FAO/WHO/UNU equation	712.4 (358.9)
*Outcomes*		Oxford (weight) equation	713.1 (340.6)
Mechanically ventilated	93 (46.7)	Oxford (weight and height) equation	714.3 (338.3)
Length of PICU stay, days	7.0 (13.0) ^#^	Talbot (weight) equation	661.5 (342.0)
*Vital signs*		Talbot (height) equation	684.7 (332.8)
Heart rate, bpm	117.6 (30.3)	Mehta equation *	463.4 (257.2)
Systolic Blood Pressure, mmHg	103.5 (18.3)		
Diastolic Blood Pressure, mmHg	61.0 (14.9)		
Body Temperature, °C	36.6 (0.7)		
Oxygen Saturation, %	97.7 (2.7)		
*Blood values*			
Hemoglobin, mg/dl	9.9 (1.8)		
Blood glucose, mg/dl	106.4 (37.3)		
C-Reactive Protein, mg/dl	2.3 (6.7) ^#^		

Abbreviations: VO_2_ = Oxygen Consumption; VCO_2_ = Carbone Dioxide Production; RQ = Respiratory Quotient; BMI = Body Mass Index; PICU = Pediatric Intensive Care Unit; REE = Resting Energy Expenditure; FAO = Food and Agriculture Organization; WHO = World Health Organization; UNU = United Nation University. Data are presented as mean and standard deviation or frequency and percentage. * mechanically ventilated children. ^#^ data are expressed as median and interquartile range (IQR) according to their distribution.

**Table 5:** Fitting performances of true REE by the methods under study.

Overall Group (*N* = 199), Measured REE = 632.3 (339.1)									
FITTING METHOD	Predicted REE		Absolute Error	Accuracy	Relative Error	Accuracy	F-Test Two-Sample		
	Mean	SD	Mean	%	Mean	%	F-Statistic	*p*-Value (Two Tails)	Pearson (R^2^)
ANN with gas	631.0	331.3	23.3	96.3	0.050	95.0	1.053	0.718	0.968
ANN with VCO_2_	637.3	332.5	65.6	89.6	0.126	87.4	1.046	0.754	0.921
ANN with VCO_2_ (ventilated)	553.1	288.6	66.4	88.0	0.144	85.6	1.101	0.647	0.866
ANN without gas	628.4	312.5	111.7	82.3	0.212	78.8	1.183	0.237	0.808
Harris–Benedict	833.5	261.6	245.4	61.2	0.603	39.7	1.680	<0.0001	0.529
Harris–Benedict for infants	718.9	356.8	182.4	71.2	0.370	63.0	0.903	0.474	0.623
Schofield (weight)	711.5	352.5	155.4	75.4	0.310	69.0	0.853	0.265	0.725
Schofield (weight and height)	712.6	350.9	151.9	76.0	0.307	69.3	0.938	0.654	0.735
FAO/WHO /UNU	712.4	357.9	160.1	74.7	0.317	68.3	0.897	0.446	0.715
Oxford (weight)	713.1	339.7	155.0	75.5	0.312	68.8	0.996	0.979	0.722
Oxford (weight and height)	714.3	337.5	150.5	76.2	0.306	69.4	1.010	0.946	0.737
Talbot (weight)	661.5	341.1	132.7	79.0	0.264	73.6	0.988	0.933	0.751
Talbot (height)	681.2	333.4	136.0	78.4	0.274	72.6	0.985	0.913	0.758
Mehta *	463.4	257.2	90.8	83.5	0.164	83.6	1.386	0.647	0.901

Abbreviations: ANN = Artificial Neural Networks; VO_2_ = Oxygen Consumption; VCO_2_ = Carbone Dioxide Production; RQ = Respiratory Quotient; REE = Resting Energy Expenditure; FAO = Food and Agriculture Organization; WHO = World Health Organization; UNU = United Nation University. * mechanically ventilated children. Measured REE in mechanically ventilated children was 543.9 (298.2).

**Table 6:** Results obtained in the three differential analyses relevant to the role of gas values.

Data Set 2	Baseline	1	2
Variables number of the data set	32	29	30
Gas variables	VO_2_; VCO_2_; RQ	none	VCO_2_
Variables selected by TWIST system	African	mechanically ventilated	South American
	height	male	African
	wasting mild	Asian	weight
	VO_2_	African	height
	VCO_2_	weight	BMI
	RQ	height	obesity
	*SatO2%*	BMI z-HFA wasting mild *body temperature* *SatO2%* *CRP*	VCO_2_ *Blood glucose* *CRP*
predictive accuracy	96.3%	82.3%	89.5%
mean absolute error	23.3	111.7	65.6
Person R^2^	0.968	0.808	0.921

Abbreviations: TWIST = Training with Input Selection and Testing; VO_2_ = Oxygen Consumption; VCO_2_ = Carbone Dioxide Production; RQ = Respiratory Quotient; BMI = Body Mass Index; SatO_2_% = oxygen saturation; z-HFA = z-score height for age; CRP = C-reactive protein. Variables only included in Data set 2 and selected by TWIST are presented in *Italic*.

## Data Availability

The dataset analyzed during the current study is available from the corresponding author on reasonable request.

## Supplementary Materials

The following are available online at https://www.mdpi.com/article/10.3390/nu13113797/s1, Additional File S1: Correlations between the original study variables and the REE value from Data set 2; Additional File S2: Real REE approximation with predictive equations from Data set 2

## Abbreviations

REE: Resting Energy Expenditure; IC: Indirect Calorimetry; VO_2_: Oxygen consumption; VCO_2_: Carbon dioxide production; RQ: Respiratory Quotient; LOS: Length of stay; ICU: Intensive Care Units; PICU: Pediatric Intensive Care Unit; WHO: World Health Organization; BMI: Body Mass Index; WFA: Weight for age; WFL: Weight for length/height LFA: Length/height for age (LFA); Hb: Hemoglobin; CRP: C-reactive protein; SatO_2_%: Oxygen saturation; SBP: Systolic Blood Pressure; DBP: Diastolic Blood Pressure; ANN: Artificial Neural Networks; ANNs: Artificial Neural Network Algorithms; TWIST: Training With Input Selection and Testing; T&T: Training/testing; IS: Input Selection; GenD: Genetic Doping Algorithm; MAE: Mean Absolute Error.
