# Supplementary material for: Artificial Neural Network Algorithms to Predict Resting Energy Expenditure in Critically Ill Children
Source: Nutrients. 2021 Oct 26;13(11):3797. doi: 10.3390/nu13113797 (PMC8618974; doi:10.3390/nu13113797)
Supplement: Supplementary file 1 [file nutrients-13-03797-s001.zip › nutrients-1402804-supplementary.pdf]

Supplementary Materials

Additional File S1 (PNG format)

Additional File S1 shows the correlations between the original study variables and the REE value from Data set 2.

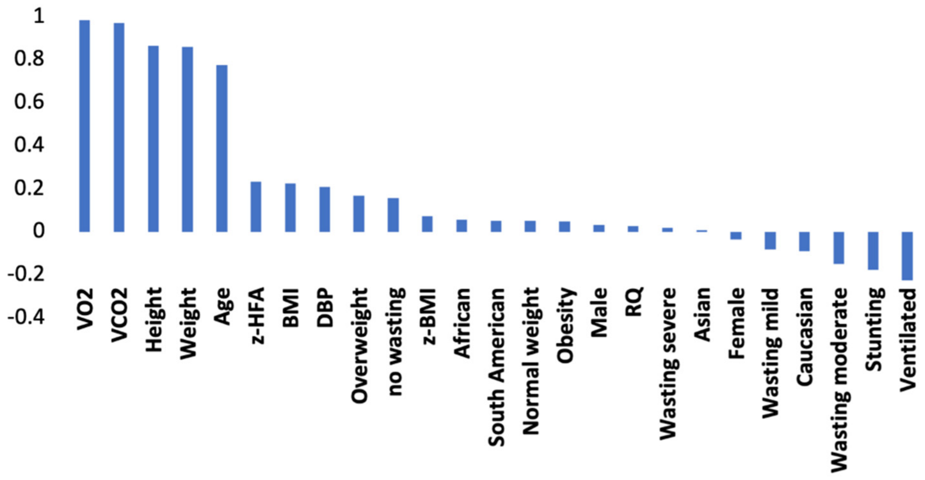

Additional File S1. Correlations between the original study variables and the REE value from Data set 2. Legend. Abbreviations: VO_2= Oxygen Consumption; VCO_2= Carbone Dioxide Production; RQ= Respiratory Quotient BMI= Body Mass Index. z-BMI= z-score BMI; z-HFA= z-score Height for Age.

Additional File S2 (PNG format)

Additional File S2 shows the Real REE approximation with predictive equations from Data set 2

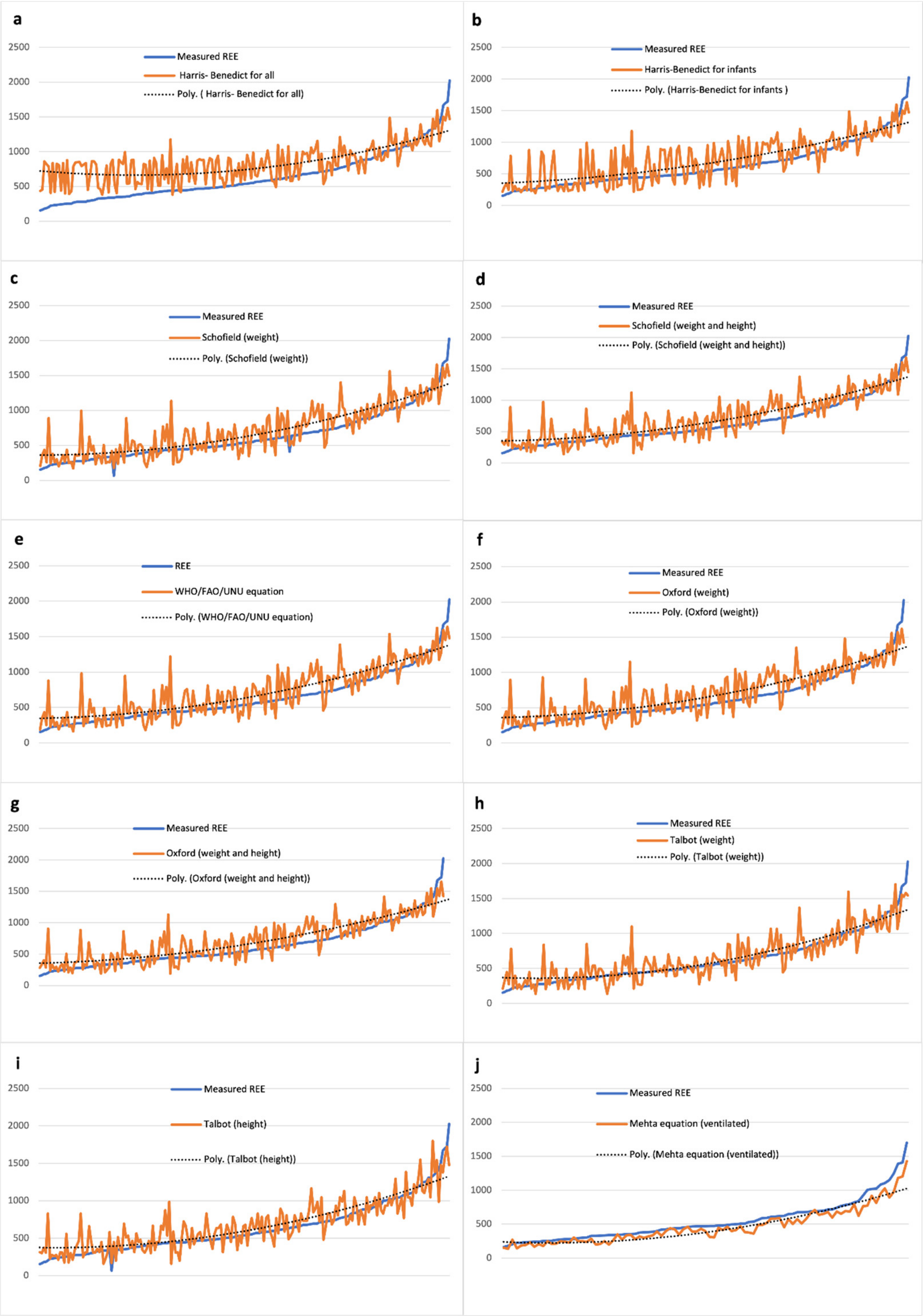

Additional File S2. Real REE approximation with predictive equations from Data set 2. Legend. Harris-Benedict for all (**a**), Harris-Benedict for infants (**b**), Schofield (weight) (**c**), Schofield (weight and height) (**d**), WHO/FAO/UNU equation (**e**), Oxford (weight) (**f**), Oxford (weight and height) (**g**), Talbot (weight) (**h**), Talbot (height) (**i**), Mehta equation in ventilated children (**j**).
